# Neuropsychomotor development in children born preterm at 6 and 12 months of corrected gestational age

**DOI:** 10.1590/1984-0462/2022/40/2020199

**Published:** 2021-09-01

**Authors:** Nathália Faria de Freitas, Cynthia Ribeiro do Nascimento Nunes, Thalyta Magalhães Rodrigues, Gislene Cristina Valadares, Fernanda Lima Alves, Caio Ribeiro Vieira Leal, Natália Maria Câmara da Luz, Marina de Oliveira Rabello, Marcia Gomes Penido Machado, Maria Candida Ferrarez Bouzada

**Affiliations:** a Universidade Federal de Minas Gerais, Belo Horizonte, MG, Brazil.

**Keywords:** Child development, Developmental disabilities, Infant, premature, Preterm, Risk factors, Desenvolvimento infantil, Deficiências do desenvolvimento, Recém-nascido prematuro, Pré-termo, Fatores de risco

## Abstract

**Objective:**

To assess the incidence of neuropsychomotor developmental delay at 6 and 12 months of corrected gestational age in children born at 32 gestational weeks or less.

**Methods:**

A descriptive and prospective study was carried out at two public maternity hospitals. Between April 2017 and January 2019, we assessed 133 children without any known risk factors for neuropsychomotor developmental delay. The Bayley III scale was used to evaluate cognitive and motor development. The p value of the numerical variables was calculated using the Mann-Whitney test, whereas proportions of categorical variables were compared using the Z-test.

**Results:**

The mean maternal age was 26±6.9 years,78.8% were from middle and lower economic classes, and 57.1% of the analyzed children were female. Children presented with a higher incidence of delay at 12 months than at 6 months (10.3 and 2.3% at 12 and 6 months, respectively, for the cognitive score; 22.7 and 12% at 12 and 6 months, respectively, for the composite motor score; and 24.7 and 8.4% at 12 and 6 months, respectively, for the fine motor score).

**Conclusions:**

Cognitive and motor developmental delays were significant, with the highest incidence at 12 months. The results of this study encourage further research on this topic, since the exclusion criteria were comprehensive and the delays in neuropsychomotor development were significant.

## RESUMO

**Objetivo:**

Conhecer a incidência de atraso de desenvolvimento neuropsicomotor de crianças nascidas com idade gestacional menor ou igual a 32 semanas aos 6 e 12 meses de idade gestacional corrigida.

**Métodos:**

Estudo descritivo e prospectivo realizado em duas maternidades públicas. Foram incluídas 133 crianças no período de abril de 2017 a janeiro de 2019 sem fatores conhecidos para atraso no desenvolvimento neuropsicomotor. Para avaliação do desenvolvimento cognitivo e motor, utilizou-se a escala Bayley III. Para calcular o valor p das variáveis numéricas, foi usado o teste de Mann-Whitney, e para as categóricas, o teste Z de comparação de proporções.

**Resultados:**

A média da idade materna foi de 26±6,9 anos, a classificação econômica prevalente foi classe média e baixa, com percentual de 78,8%, e os recém-nascidos do sexo feminino representaram 57,1% das crianças. As crianças apresentaram maior incidência de atraso aos 12 meses, sendo 10,3% aos 12 meses e 2,3% aos 6 meses para o escore cognitivo, 22,7 e 12% aos 12 e 6 meses, respectivamente, no escore motor composto e 24,7% aos 12 meses e 8,4% aos 6 meses no escore motor fino.

**Conclusões:**

Atraso nos desenvolvimentos cognitivo e motor foi significativo, com maior incidência aos 12 meses. O escore motor composto apresentou a maior incidência de atraso de desenvolvimento. Os resultados do estudo são instigantes, uma vez que os critérios de exclusão foram abrangentes, e os atrasos no desenvolvimento neuropsicomotor, significativos.

## INTRODUCTION

Neuropsychomotor development is a process of physical and neurological changes. It begins at conception and involves biological, social-emotional and psychic aspects for the construction of brain architecture.^1^ ^,^ ^2^ It can be understood as a vital process involving several factors, such as physical growth, followed by neurological, behavioral, cognitive and social-emotional maturation of the child.^3^

The first years of life of a child are considered as essential for the construction of a solid base for development throughout life.^2^ Therefore, a damaged initial development can interfere in the health of the individual and, consequently, trigger cognitive disabilities, learning impairment, language problems, behavioral and language disorders.^4^

Adverse factors, such as prematurity, can change the evolution of neurological development and trigger a delayed neuropsychomotor development.^4^ Around the world, prematurity is the main cause of child mortality until the age of 5 years. The estimation is that, per year, one million small and sick newborns survive with a long-term disability, including cerebral palsy and cognitive delays.^5^

Developmental delays are triggered as a product of genetic, biological, psychological and environmental risk factors, and the cumulative effect of these factors can cause bigger problems.^6^ In this context, neuropsychomotor development studies in preterm newborns (PTNB) show significant results in the incidence of developmental delay;^7^ ^–^ ^9^ however, minor problems in PTNB can present better outcomes in neuropsychomotor development. Therefore, the question is: what is the incidence of neuropsychomotor developmental delay in children whose gestational age was less than 32 or 32 weeks at 6 and 12 months of corrected gestational age?

The early identification of changes and intervention can minimize the negative effect of future problems in a child’s life.^2^ Therefore, knowing the incidence of neuropsychomotor developmental delay in the selected population of children born preterm may indicate best practices, both in the neonatal period and in the follow-up of PTNB. The objective of the study was to know the incidence of neuropsychomotor developmental delay in children whose gestational age was less than 32 or 32 weeks, at 6 and 12 months of corrected gestational age (CGA).

## METHOD

**Figure 1:**
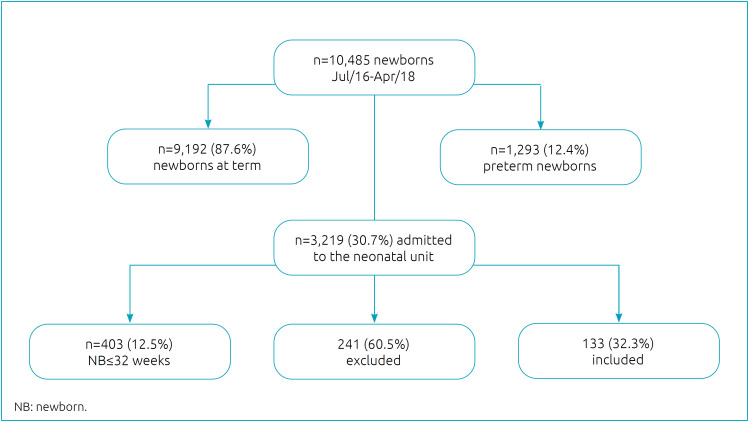
Children included in the study from July 2016 to April 2018, Belo Horizonte, Minas Gerais, Brazil.

This is a descriptive and prospective study carried out from July, 2016, to January, 2019, in children born at two public maternity hospitals that are reference for high risk cases in the city of Belo Horizonte (MG). We included 133 children whose gestational age was less than 32 or 32 weeks (Figure 1).

The exclusion criteria were: Apgar lower than 7 at 5 minutes of life, congenital malformations and/or genetic syndromes, vertically transmitted infections, peri-intraventricular hemorrhage (PIVH) grades III and IV, and leukoencephalomalacia, infection of the central nervous system, severe bronchopulmonary dysplasia, mothers and newborns who died during hospitalization or after hospital discharge, mothers that did not live in the metropolitan region of Belo Horizonte, mothers with a history of drug use during pregnancy, and children suspected of autism. Exclusion criteria were wide, in order to select a sample of PTNB with lower risks of changes in neurodevelopment.

Maternal and neonatal data were revised since birth until hospital discharge, including neonatal and therapeutic variables, complications or adverse events that could affect the result of neurodevelopment. The criterion to determine gestational age was based on the obstetric ultrasound performed until the 12^th^ week of pregnancy. The socioeconomic profile evaluation was classified according to the Economic Classification Criteria in Brazil.^10^

After hospital discharge, the children returned to the outpatient clinics of the maternity hospitals for neuropsychomotor evaluations using the Bayley III scale of infant and toddler development,^7^ which was applied at 6 and 12 months of CGA. The Bayley III scale includes cognitive, motor, linguistic, social-emotional evaluation, and is the most used instrument for PTNB.^2^ It is among the best ones to assess child development, being acknowledged around the world for the ability to provide a broad assessment of child development.^11^ In Brazil, the Bayley III scale was transculturally translated and adapted, and is nowadays available for use.^12^

In the development assessment, two categorical variables were defined for each one of the cognitive and motor scales, and the normal composite score was considered equal to or higher than 85 (normal or accelerated development). The score was altered when lower than 85 (mild, moderate or significantly delayed development) in the Bayley III scale.^11^ The scaled value was considered for cognitive, fine and gross motor lower than or equal to 6 (altered development), and higher than or equal to 7 (normal or superior development).^11^ The children with changes in evaluation were referred to specialized care, with a descriptive report about the clinical evaluation of growth and neuropsychomotor development. The evaluation was performed by the same researcher after being trained to use the scale, at the constant presence of an observer researcher. The children were included in the investigation after the parents or tutors signed the Informed Consent Form, approved by the Research Ethics Committee, report n. 1.577.657.

The database was organized in an Excel file, with independent double typing. After the verification for errors and inconsistencies, the analysis was executed using the Statistical Package for the Social Sciences (SPSS) software, version 18 for Windows and MiniTab 17. In the descriptive statistical analysis of the categorical variables, we obtained absolute and relative frequencies, and calculated the means and standard deviation for the symmetrical variables (p>0.05). The medians and 25 and 75 percentiles were calculated for asymmetrical variables (p<0.05). The normality distribution of continuous variables was investigated by the Shapiro-Wilk test. To calculate the p-value of numerical variables, we used the Mann-Whitney test and, for categorical variables, a two-proportion Z-test. The statistical analyses were considered significant when ≤0.05.

## RESULTS

We assessed 133 children at 6 months and, of these, 97 returned at 12 months of CGA, after the period of data analysis (Figure 1). Mean maternal age (±standard-deviation) was 26 years (±6.99); 97.7% of the pregnant women attended prenatal care; the prevalent economic status was middle and low class; and 78.8 and 60.9% of the mothers had concluded high school. Regarding life habits, 6.8% of the mothers declared to be smokers. Among the pregnant women, 53.4% presented with some comorbidity, and systemic arterial hypertension was the most prevalent one (37.1%).

Female newborns were more frequent – 57.1%. We measured the child growth parameters at birth using the Intergrowth-21^st^ charts,^13^ and the follow-up was based on corrected age in the child’s passbook chart (Table 1).

**Table 1:** Variables related to gestational age and anthropometric data at birth, discharge, 6 and 12 months of corrected gestational age in newborns with gestational age ≤32 weeks*.

	Gestational age (weeks)	Weight (g)	Head Circumference (cm)	Length (cm)
Birth	30.6 (29–31.8)	1,250 (1,037–1,552)	27 (25–28.5)	39 (36–41)
Discharge	36.1 (35.3–37.6)	2,012 (1,837–2,380)	31.8 (30.9–33)	44 (43–6)
Six months CGA	–	6,950 (6,315–7,800)	43 (42–44)	65 (62–66,5)
12 months CGA	–	8,905 (8,032–10,000)	46 (44.8–47)	73 (70–76)

* All data are presented in median (25–75 percentile); CGA: corrected gestational age.

In the evaluation of outpatient follow-up, 74% of the children were assessed by a multidisciplinary group, and 62.4% of the children at 6 months and 67% at 12 months of CGA became ill; respiratory conditions were the most prevalent ones.

For categorical variables, in cognitive and composite motor development and scaled profile at 6 and 12 months of CGA, we observed a higher incidence of children with delay at 12 months, when compared to those at 6 months (p≤0.005), and statistically significant difference between the assessed months for cognitive (p=0.017), fine motor (p=0.001) and composite motor (p=0.047) scores. The gross motor score did not present statistical differences (p=0.530) (Table 2).

**Table 2:** Evaluation of cognitive development and composite motor and scaled profile in the Bayley III scale at 6 and 12 months of corrected gestational age.

Scale	Classification	6 months n (%)	12 months n (%)	p-value
Composite cognitive	Normal (≥85)	130 (97.7)	87 (89.7)	
	Delay (<85)	3 (2.3)	10 (10.3)	0.017
Composite motor	Normal (≥85)	117 (88.0)	75 (77.3)	
	Delay (<85)	16 (12.0)	22 (22.7)	0.047
Cognitive	Normal (≥7) Delay (≤6)	130 (97.7) 3 (2.3)	87 (89.7) 10 (10.3)	0.017
Fine motor	Normal (≥7) Delay (≤6)	122 (91.6) 11 (8.4)	73 (75.3) 24 (24.7)	0.001
Gross motor	Normal (≥7) Delay (≤6)	120 (90.1) 13 (9.9)	85 (87.6) 12 (12.4)	0.530

In numerical variables, for cognitive, fine motor and composite motor scores we observed better results at 6 months in comparison to 12 months, and a statistically significant difference between the assessed months for cognitive (p<0.001), fine motor (p<0.001) and composite motor (p<0.001) scale. The gross motor variable did not show any statistical difference (p=0.084) (Table 3).

**Table 3:** Mean of evaluation scores in the Bayley III scale at 6 and 12 months of corrected gestational age.

Scale	6 months Median (p25–p75)	12 months Median (p25–p75)	p-value
Composite cognitive	100 (95–105)	95 (85–100)	<0.001
Composite morot	97 (85–107)	91 (85–97)	<0.001
Cognitive	10 (4–13)	9 (1–15)	<0.001
Fine motor	10 (4–14)	8 (1–14)	<0.001
Gross motor	9 (1–16)	9 (2–18)	0.084

## DISCUSSION

In this study, we observed an increasing percentage of neuropsychomotor developmental delay in the 12-month period, when compared to 6 months of CGA. Besides, it was possible to verify that the composite motor score presented higher incidence of delay when compared to composite cognitive score, both at 6 months and 1 year of CGA. In the evaluations of 6 and 12 months of CGA, the delay was significant in cognitive (p=0.017), composite motor (p=0.047), and fine motor (p=0.001) function.

The identification of higher developmental delay at 12 months of CGA in comparison to 6 months, besides the higher incidence of motor delay, can be associated with subtle delays in neurodevelopment, and the consequent underestimation of changes that decreased with age in the evaluation.^14^ Besides, developmental delays, especially cognitive ones, could not even be identified at this age group; such an identification is only possible among preschoolers,^15^ once the first year of life is the period when children face higher demands.^16^ However, it is important to consider that, despite the fact that children included in the study were from the group with fewer vulnerabilities, prematurity is still one of the factors that can cause neurodevelopmental impairment, regardless of associated events.^4^ Studies of neuropsychomotor development,^7^ ^,^ ^8^ ^,^ ^17^ ^,^ ^18^ especially in the first year of life of children born preterm, have shown damage in cognitive and motor functions.

Cognitive disorders and structural and functional disabilities throughout the life of PTNB have been associated with changes in white and grey matter and cortical areas, which is a result of episodes of ischemic hypoxia, injuries to the germinal matrix, and, as a consequence, hypomyelination and diffuse axonal injury.^19^ ^,^ ^20^ Results of an investigation about the relationship between a magnetic resonance examination in PTNB younger than 30 weeks and early motor development show that, in children assessed with Bayley III at 6 months of CGA, low motor scores were related to the reduction of subcortical grey matter.^21^ Due to preterm birth, the neurodevelopmental process of the cells has flaws in the stage of organization and delay in the loss of non-functional synapses, which is an important fact to define the pathways of communication between the brain regions.^22^ Considering that the first year of life is the period when the brain grows the most, and faster, which therefore leads to the increase of neural connections,^23^ the cognitive and motor skills acquired at this stage can be compromised.

When analyzing studies about neurodevelopment, it was possible to observe that the exclusion criteria used in our study were rigorous and comprehensive — 59.8% of the newborns with 32 or fewer weeks of gestational age were not eligible for not meeting the inclusion criteria. The exclusion percentage of children was significant, however, it is worth to mention that the locations where the study was carried out are reference in high risk pregnancies; therefore, more severe mother and child health conditions are referred to them. It was necessary to know the aspects of neurodevelopment in a population of PTNB that was less vulnerable to changes in neuropsychomotor development, and to identify if even a PTNB with good clinical evolution in the neonatal period and after hospital discharge presented with developmental delays in the first year of life. Therefore, the risk of bias was minimized, especially in case of severe morbidities that would cause damage to child development, such as PIHV grades III and IV and congenital malformations. Considering the rigorous criteria to admit children in the study, the results were close to those found in the literature, despite the impossibility to compare them with previous results.

In this sense, a similar study in PTNB with comprehensive inclusion criteria, such as in this study, showed that 18% of the children assessed with Bayley III at 7 months of CGA presented only with composite motor delay.^18^ The median (p25–p75) of gestational age was 33 weeks (25-36), however, a relevant aspect that is different from this study is that 65.5% of the included newborns were late premature infants (34 to 36 weeks of gestational age), which could explain the findings regarding the outcome of cognitive development. Eickmann et al. assessed children at 6 and 12 months of CGA using the Bayley III scale. The presence of infection and/or congenital malformations and genetic syndromes was considered as an exclusion criterion, and mean gestational age in the PTNB group was 33 weeks. In the evaluated period, the mean (±standard deviation) score in the composite cognitive domain was 11.06 (±9.1); for the composite motor domain, 106 (±11.4); for the fine motor domain, 11.5 (±2.4); and for the gross motor domain, 10.5 (±2.5). The percentage of neuropsychomotor developmental delay and the scaled cognitive score were not presented.^17^

A study using Bayley III and the development of infants in the first year of life revealed a mean of composite cognitive delay of 6%; composite motor delay, 22%; fine motor delay, 12%; and gross motor delay, 47%.^9^ Likewise, in a performance study including PTNB younger than 27 weeks, followed up from 2 months to 2.5 years of age, in comparison with at term newborns and using the Bayley III scale, the prevalence of neuropsychomotor developmental delay was 10.8% in the composite cognitive scale; 12.4% for fine motor; and 7% for gross motor functions. The score and percentage of composite motor delay were not presented.^8^ In both studies, no exclusion criteria was used. The differences in percentage can be explained by the age groups in the first year of life, the type of study and the defined exclusion criteria.

On the other hand, the incidence of delay was significantly higher in a prospective study with children born with gestational age lower than or equal to 32 weeks at 12 months of CGA, using the Bayley III scale. The proportion of composite cognitive delay was 25%; composite motor delay, 35%; fine motor delay, 35.8%; and gross motor delay, 43.2%.^7^ The difference in the findings, in comparison to our study, can be related to the absence of exclusion criteria, and biological and environmental conditions can affect the development of children. Greene et al. did not use exclusion criteria in the sample of children with extreme low weight assessed in the first year of life with 8 to 12 months of CGA. The mean composite cognitive delay was 6%, and in composite motor delay, 22%; in motor fine delay, 12%, and in gross motor delay, 47%.^9^ Another recent study in a follow-up outpatient clinic evaluated 120 children born with gestational age lower than or equal to 32 weeks at 12 months of CGA, using the Bayley III scale. The proportion of composite cognitive delay was 25%; in composite motor delay, 35%; in fine motor delay, 35.8%; and in gross motor delay, 43.2%. No newborn with risk factors was excluded.^7^ It is possible that the absence of exclusion criteria be associated with the high incidence of delays.

Prematurity has been mentioned as the cause of major biological risk in cognitive and motor delays that can affect the development of the child.^24^ In case of preterm infants, neuropsychomotor development works in an immature brain associated to environmental factors that can result in significant cognitive and motor changes.^17^ Environmental risks, socioeconomic status and schooling of the parents are some of the risk factors for neuropsychomotor development.^4^ In this study, the child belonging to middle and low classes (classes C/D/E) was a factor for higher neuropsychomotor developmental delay (78.8%). In a study to assess the prevalence of of neuropsychomotor developmental delays of premature infants, it is suggested that belonging to middle and low socioeconomic classes was associated to lower cognitive scores.^25^ On the other hand, in the present investigation, 60.9% of the mothers concluded high school, and maternal schooling has been related as an essential factor for neuropsychomotor development;^26^ and the female gender (57.1%) presents better developmental scores.^27^

In the evaluation of neurodevelopment in PTNB in the first year of life, it is possible to observe that the type of study, the eligibility criteria and the period of the evaluations directly interfere in the obtained results. Therefore, it is not possible to compare our findings with those from previous studies. In fact, it was possible to identify that cognitive and motor developmental delays were significant at 6 and 12 months of CGA, even in a population of selected PTNB; the occurrence of aggravation cannot be excluded. On the other hand, it is believed that the incidence of changes could have been higher in case the children had not been included in a selective manner.

These findings reinforce that, for better outcomes in the neuropsychomotor development of preterm newborns in the first year of life, early intervention since birth associated with specific follow-up programs is an essential strategy to identify morbidities.^28^ In this sense, the PTNB hospitalized in neonatal units and the tutors should be encouraged to an affectional and cognitive exchange that goes beyond care and nutrition of the newborn, sustained by the kangaroo method.^29^ When exposed to the kangaroo position, newborns present with increased concentrations of oxytocin, which influence synaptic plasticity, favoring the growth of the brain.^30^ Early intervention has a positive impact on cognitive and motor outcomes in childhood.^31^ The follow-up of the individual after hospital discharge, associated with a multidisciplinary approach, can minimize the incidence of neuropsychomotor developmental delay in the first year of life of preterm infants.^28^

The study’s limitation is the fact that we did not identify, in this population, children who hunderwent frequent physical therapy, occupational therapy and speech language therapy interventions, even though they were all followed-up in outpatient clinics. Another limitation was the fact that the study was descriptive, not making a cause and effect relationship. However, despite these limitations, its exploratory character enables to subsidize neuropsychomotor developmental studies, especially in the first year of life, and to reflect on more effective health public policies, once the exclusion criteria were comprehensive and, even so, cognitive and motor developmental delays were significant.

The conclusion was that children born with gestational age lower than or equal to 32 weeks presented increased percentage of neuropsychomotor developmental delay at 12 months when compared to those at 6 months of CGA. Composite motor score presented higher incidence of delay when compared to composite cognitive score. More studies are necessary to conduct a better analysis of perinatal, environmental, and social-emotional aspects that can be associated with neuropsychomotor development.
